# Anti-ganglioside antibodies are removed from circulation in mice by neuronal endocytosis

**DOI:** 10.1093/brain/aww056

**Published:** 2016-03-26

**Authors:** Madeleine E. Cunningham, Rhona McGonigal, Gavin R. Meehan, Jennifer A. Barrie, Denggao Yao, Susan K. Halstead, Hugh J. Willison

**Affiliations:** Neuroimmunology Group, Institute of Infection, Immunity and Inflammation, College of Medical, Veterinary and Life Sciences, University of Glasgow, UK

**Keywords:** anti-ganglioside antibody, motor nerve terminal, neuropathy, autoantibody, endocytosis

## Abstract

**See van Doorn and Jacobs (doi:10.1093/brain/aww078) for a scientific commentary on this article.  **

In axonal forms of Guillain-Barré syndrome, anti-ganglioside antibodies bind gangliosides on nerve surfaces, thereby causing injury through complement activation and immune cell recruitment. Why some nerve regions are more vulnerable than others is unknown. One reason may be that neuronal membranes with high endocytic activity, including nerve terminals involved in neurotransmitter recycling, are able to endocytose anti-ganglioside antibodies from the cell surface so rapidly that antibody-mediated injury is attenuated. Herein we investigated whether endocytic clearance of anti-ganglioside antibodies by nerve terminals might also be of sufficient magnitude to deplete circulating antibody levels. Remarkably, systemically delivered anti-ganglioside antibody in mice was so avidly cleared from the circulation by endocytosis at ganglioside-expressing plasma membranes that it was rapidly rendered undetectable in serum. A major component of the clearance occurred at motor nerve terminals of neuromuscular junctions, from where anti-ganglioside antibody was retrogradely transported to the motor neuron cell body in the spinal cord, recycled to the plasma membrane, and secreted into the surrounding spinal cord. Uptake at the neuromuscular junction represents a major unexpected pathway by which pathogenic anti-ganglioside antibodies, and potentially other ganglioside binding proteins, are cleared from the systemic circulation and also covertly delivered to the central nervous system.

## Teaser

How does anti-ganglioside autoantibody clearance from the circulation affect their detection and neurotoxicity? Cunningham *et al*. describe an uptake pathway for these autoantibodies at motor nerve terminals and their delivery into the brain parenchyma. This highlights the limitations of serum antibody measurements and suggests a possible entry mechanism for their central effects.

## Graphical abstract

**Figure:**
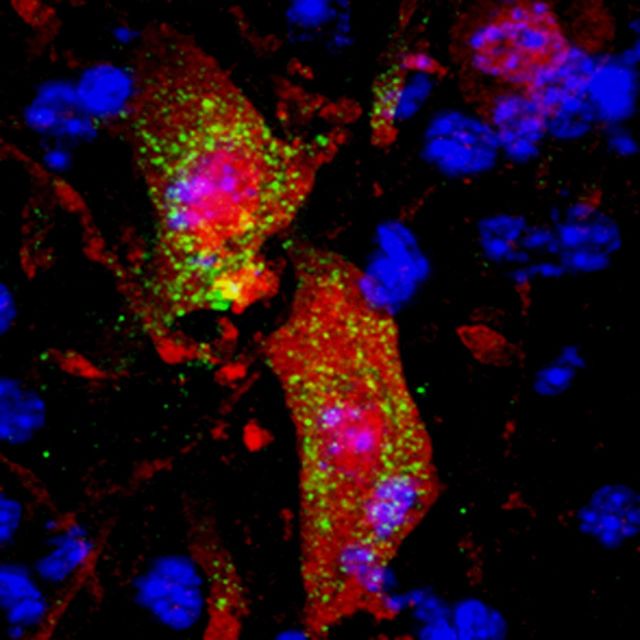
**See van Doorn and Jacobs (doi:10.1093/brain/aww078) for a scientific commentary on this article.  ** How does anti-ganglioside autoantibody clearance from the circulation affect their detection and neurotoxicity? Cunningham *et al*. describe an uptake pathway for these autoantibodies at motor nerve terminals and their delivery into the brain parenchyma. This highlights the limitations of serum antibody measurements and suggests a possible entry mechanism for their central effects.

## Introduction

The Guillain-Barré syndromes are autoimmune neuropathies in which immune factors acutely injure nerves (van den Berg *et al.*, 2014). In axonal forms of Guillain-Barré syndrome, neurotoxic anti-ganglioside antibodies (AGAbs) bind to gangliosides on exposed axolemmal membranes where they fix complement (Plomp and Willison, 2009). Gangliosides are ubiquitous, yet very highly enriched in neurons and their projecting axons, including the presynaptic neuromuscular junction (NMJ), residing in the outer leaflet of the plasma membrane facing the extracellular milieu of the synaptic cleft (Schengrund, 2015). The NMJ is a major site of AGAb binding but is relatively resistant to complement-mediated injury due to rapid internalization of antibody by endocytosis, compared with the endocytically inactive node of Ranvier, which is highly vulnerable to injury (Fewou *et al.*, 2012). Conversely, clostridial neurotoxins require endocytic uptake to mediate their neurotoxicity (Rummel, 2013). Similarly, endocytic uptake is a major internalization pathway for growth factors, antibodies and viruses (Ritchie *et al.*, 1986; Fewou *et al.*, 2014; Schengrund, 2015). Herein we analysed the effect of neuronal endocytic uptake on clearance of AGAb from the systemic circulation.

## Materials and methods

### Mice

Male and female wild-type, GalNAcT^−/−^ and GalNAcT^−/−^-*Tg(neuronal)* were used at 6–8 weeks (for *in vivo*) or 8–12 weeks of age (for *ex vivo)*. Mice of different genotypes were age-matched and where possible, littermate controls were used. For *ex vivo* studies, mice were cross-bred with B6.Cg-Tg(Thy1-CFP/S100B-GFP) transgenic mice, expressing cyan fluorescent protein (CFP) and green fluorescent protein (GFP) in their axons and Schwann cells, respectively (Feng *et al.*, 2000; Zuo *et al.*, 2004). All procedures were conducted in accordance with the United Kingdom Animals (Scientific Procedures) Act of 1986.

### Anti-ganglioside antibodies and normal human serum

All AGAbs used were generated previously by immunization of ganglioside-deficient mice with ganglioside liposomes or ganglioside-mimicking *Campylobacter jejuni* lipo-oligosaccharide as described previously (Bowes *et al.*, 2002; Boffey *et al.*, 2005). The origin and properties of these antibodies have previously been reported (Greenshields *et al.*, 2009). Normal human serum as a source of human complement was taken from a single donor and stored in aliquots at −80 °C.

### *Ex vivo* internalization studies

Triangularis sterni muscle was dissected and mounted in Ringer’s solution as described previously (McGonigal *et al.*, 2010). Muscles were labelled with AGAb (100 µg/ml) plus 2 µg/ml alpha-bungarotoxin (BTx, Alexa Fluor® 488 or 647) (Molecular Probes) for 30 min at 4 °C. Temperature-dependent internalization studies were performed as previously described (Fewou *et al.*, 2012), using wild-type, GalNAcT^−/−^ and GalNAcT^−/−^-*Tg(neuronal)* triangularis sterni with secondary antibodies applied overnight.

For stimulation-dependent internalization studies, following initial AGAb labelling and washing steps, intercostal nerves were stimulated with a Grass SD9 square pulse stimulator (Natus Medical Inc) using platinum electrodes (5 s at 10 Hz, 30 V with a pulse duration of 0.3 ms) before fixation for immunohistology. Botulinum toxin A (BoNT/A) was added in one experimental group to demonstrate that blocking synaptic vesicle docking prevents the uptake of AGAb in response to stimulation. Details can be found in the Supplementary material.

To assess whether rapid AGAb uptake induced by synaptic activity resulted in protection of the nerve terminal from complement-mediated injury, triangularis sterni muscles were treated with 40% normal human serum in Ringer’s solution for 30 min at 37 °C. Membrane attack complex (MAC) was identified as previously described (Halstead *et al.*, 2008).

### *In vivo* anti-ganglioside antibody clearance studies

Following a baseline blood sample taken 1 day prior, mice were administered 250 μg of AGAb intraperitoneally. Further blood samples were taken by tail vein venesection on Days 1, 3, 6 and upon sacrifice. Cervical cord (C4-C6) were removed and post-fixed in 4% paraformaldehyde overnight at 4 °C, then in 30% sucrose overnight at 4 °C. Details of staining procedures are provided in the Supplementary material.

Full details of exercise studies are provided in the Supplementary material

### Image acquisition and quantitation

Images for analysis of fluorescent intensity were captured using an LSM 5 Pascal confocal microscope and image analysis was performed using ImageJ software as previously described (Greenshields *et al.*, 2009). Illustrative images were captured using a Zeiss AxioImager Z1 microscope with ApoTome attachment. For antibody intensity measurements in triangularis sterni preparations, 15 images were taken per triangularis sterni. For spinal cord, nine images were captured per mouse.

### Ganglioside liposome immunization

Liposomes were prepared as per previously described (Bowes *et al.*, 2002), stored at 4 °C until used and kept for up to 5 days. Immunizations were carried out using the protocol described in Fig. 3.

### Enzyme-linked immunosorbent assays

To test for anti-ganglioside antibody reactivity in serum, ELISAs were performed as per previously described protocol (Greenshields *et al.*, 2009). Sera from mice were applied at 1/100 dilution.

### Glycoarray

Using combinatorial glycoarray (Rinaldi *et al.*, 2009), sera and monoclonal antibodies were tested against single lipids and lipids complexes using a procedure that was modified for use with fluorescence readout, as previously described. Full details of the protocol are provided in the Supplementary material.

### ELISpots

To quantitatively determine ganglioside antigen-specific antibody responses in mice that were immunized with ganglioside liposomes, ELISpots were performed using splenocytes. The detailed ELISpot protocol is provided in the Supplementary material.

### Experimental design

Mice were assigned to each experiment depending on the availability of age-matched mice across genotypes. Mice were coded and assigned to genotype groups after analysis. Sample size estimation was chosen using G*power software (v 3.1.9.2), with an α-error of 0.05 and a power of 0.8. For *in vivo* clearance studies, based on preliminary data, an effect size of 2.5 was chosen, while for antibody intensity measurements (where individual NMJs or NeuN positive cells were ranked) an effect size of 0.4 was chosen. Sample sizes also corresponded with previous similar studies (Greenshields *et al.*, 2009).

### Statistical analysis

All statistics were performed using GraphPad Prism 6, assuming a significance level of 0.05. Outliers of antibody intensity measurements were removed via ROUT analysis (Q = 1%). Non-parametric data were analysed by either Mann-Whitney or Kruskal-Wallis test (with Dunn’s multiple comparison test) and displayed as Tukey’s box and whisker plots showing the spread of all NMJs or NeuN positive cells analysed per condition. Details of box and whisker plots are provided in the Supplementary material. Parametric data were compared by either Student’s *t -*test or one- or two-way ANOVA (see figure legends). Contingent data were compared by Fisher’s exact test or Chi-squared test.

## Results

**Figure 1:**
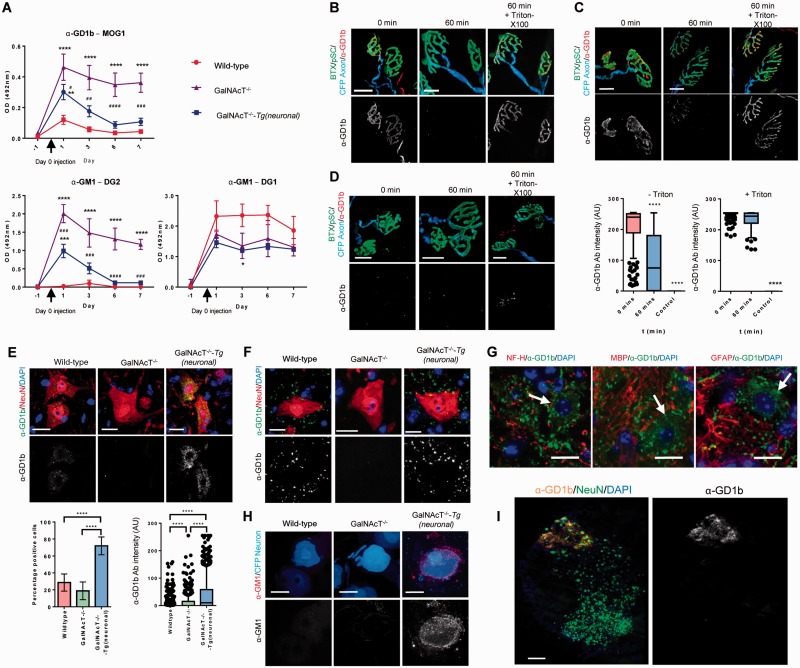
**Anti-ganglioside antibody is internalized at the motor nerve terminal and trafficked to the spinal cord.** (**A**) Serum levels of monoclonal antibodies over 7 days following i.p. injection of 250 µg. Wild-type mice (*n *= 9), which have the highest expression of ganglioside antigen clear antibody most rapidly while GalNAcT^−/−^ mice (*n *= 8) retain circulating anti-ganglioside antibody over 7 days. GalNAcT^−/−^-*Tg(neuronal)* mice (*n *= 9) show intermediate rate of clearance. Antibody that cannot bind target ganglioside in live tissue but is known to bind by ELISA is not cleared in any genotype. *Significance versus wild-type, ^#^Significance versus GalNAcT^−/−^ *P < 0.05, ^***^P < 0.001, ^****^P < 0.0001 versus wild-type. ^##^*P *< 0.01 versus GalNAcT^−/−^, two-way ANOVA with Tukey’s multiple comparison test. (**B**) Wild-type motor endplates from triangularis sterni muscle (*n *= 3 animals) were labelled at 0 min with anti-GD1b antibody, and upon incubation at 37 °C for 60 min, labelling is reduced (*middle column*). Permeabilization of the membrane with Triton™ X-100 and reprobing with secondary antibody reveals presence of antibody within the motor nerve terminal. (**C**) Illustrative images show NMJs from GalNAcT^−/−^-*Tg(neuronal)* mice (*n *= 3 animals) were labelled at 0 min with anti-GD1b antibody, but upon incubation at 37 °C, labelling is no longer present. Permeabilization of the membrane with Triton™ X-100 and reprobing with secondary antibody reveals presence of antibody within the motor nerve terminal. Graphs show quantification of antibody binding in GalNAcT^−/−^-*Tg(neuronal)* mice, where surface labelling at 60 min timepoint is significantly reduced compared to 0 min before permeabilization, but statistically similar following permeabilization and reprobing with secondary antibody. ^****^P < 0.0001 versus 0 min, Kruskal-Wallis with Dunn’s multiple comparisons test. Controls are secondary antibody only. (**D**) In GalNAcT^−/−^ mice, motor endplates (*n *= 3 animals) showed no anti-GD1b antibody binding at 0 min, nor following 60 min incubation at 37 °C. Permeabilization of the membrane with Triton™ X-100 shows no antibody presence in the nerve terminal. (**E**) One day following a 1 mg injection of anti-GD1b Ab (*n *= 3 per group), GalNAcT^−/−^-*Tg(neuronal)* mice showed presence of anti-GD1b Ab in the ventral horn neurons, which was significantly higher (^****^P < 0.0001) than both wild-type and GalNAcT^−/−^ mice by positive/negative cell counts (chi-squared test followed by multiple Fisher’s exact tests) and intensity of AGAb over NeuN staining (Kruskal-Wallis with Dunn’s multiple comparison test). (**F**) Cords taken 5 days after MOG1 injection appeared to only show antibody in the neuropil surrounding the neuron body. (**G**) Antibody localization in GalNAcT^−/−^-*Tg(neuronal)* mice spinal cord after 5 days. Antibody did not appear to be localized to axons (NF-H), myelin (MBP) or astrocytes (GFAP) when translocated from the spinal neuron. Neuron cell bodies are shown by arrows. (**H**) GalNAcT^−/−^-*Tg(neuronal)* mice show surface deposits of AGAb in some dorsal root ganglion cells 24 h after passive immunization but show negligible presence of internal AGAb. (**I**) Hemisection of mid-cervical cord of GalNAcT^−/−^-*Tg(neuronal)* mouse demonstrating the prominent distribution of AGAb in the ventral horn but not elsewhere. Scale bars: **B**–**H** = 20 μm; **I** = 200 μm. Non-parametric data are displayed as box and whisker plots.

When wild-type mice are passively immunized with monoclonal anti-GD1b IgG AGAb (250 µg intraperitoneally), we observed that the AGAb were greatly reduced in the circulation only 24 h after injection, representing much faster clearance than previously reported for antibodies of the same isotype (Zuckier *et al.*, 1989) (Fig. 1A). To demonstrate clearance was antigen-dependent, AGAbs were also passively transferred to GalNAcT^−/−^mice, which synthesize no complex gangliosides and are thus completely devoid of AGAb targets (Takamiya *et al.*, 1996). In GalNAcT^−/−^ mice, anti-GD1b AGAb levels were still highly elevated after 1 week. As ganglioside expression is widespread throughout the body, albeit enriched in the nervous system, many cell types are potentially responsible for antigen-specific AGAb clearance. To identify the specific contribution of neuronal AGAb uptake on systemic depletion of AGAb, we passively immunized mice in which GalNAcT enzyme activity, and thus complex ganglioside expression, is limited to neurons [GalNAcT^−/−^-*Tg(neuronal)*] (Yao *et al.*, 2014). In these mice, circulating anti-GD1b AGAb decayed prematurely to undetectable levels by Day 6. Thus, the contribution of neuronal clearance alone, compared to systemic AGAb depletion, is substantial.

To investigate how endocytic uptake might affect clearance of AGAbs with different avidities for membrane gangliosides, we passively immunized mice with two different anti-GM1 IgG mAbs, DG1 and DG2 previously reported in detail (Greenshields *et al.*, 2009). Importantly, DG1 binds GM1 strongly in ELISA-based immunoassays but is unable to bind GM1 in intact plasma membranes due to steric hindrance from laterally-interacting gangliosides (a principle well recognized among AGAbs) (Greenshields *et al.*, 2009; Rinaldi *et al.*, 2009). In contrast, DG2 binds GM1 strongly both in immunoassays and in plasma membranes. As predicted, the non-tissue binding AGAb DG1 remained highly elevated in the serum of all three genotypes throughout the experimental time course. In contrast, AGAb DG2 is never detectable in the circulation of wild-type mice, remains highly elevated after 7 days in the GM1-deficient GalNAcT^−/−^ mice and at intermediate levels in GalNAcT^−/−^-*Tg(neuronal)* mice (Fig. 1A). In conclusion, AGAb clearance is an antigen-specific process that requires plasma membrane ganglioside binding to occur. Furthermore, a high proportion of AGAb is cleared solely by binding to neuronal gangliosides.

From prior work we expect the NMJ to be the major neuronal uptake pathway for AGAbs (Plomp and Willison, 2009). In *ex vivo* triangularis sterni neuromuscular preparations, anti-GD1b mAb antibody bound on the surface of wild-type (Fig. 1B) and GalNAcT^−/−^-*Tg(neuronal)* NMJ plasma membranes (Fig. 1C) and was subsequently internalized within 1 h, but in contrast failed to bind (or be internalized) at the GD1b-deficient GalNAcT^−/−^ NMJ (Fig. 1D). Internalization was assessed by permeabilizing the plasma membrane with Triton™ X-100, thereby allowing secondary antibody access and thus visualization of intracellular AGAb. As ganglioside binding toxins, including tetanus and cholera toxin, are known to be retrogradely transported to the spinal cord (Stoeckel *et al.*, 1977), we then examined the ventral horns in the spinal cord of passively immunized mice at 24 h post-immunization and observed significant AGAb deposits in anterior horn cells of GalNAcT^−/−^-*Tg(neuronal)* mice and minor deposits in wild-type mice, whereas in GalNAcT^−/−^ mice no deposits were observed, as expected (Fig. 1E). By 5 days the AGAb had trafficked out of anterior horn cells into adjacent neuropil of the spinal cord (Fig. 1F). As wild-type mice have the potential to clear AGAbs through all ganglioside-expressing plasma membranes, these data suggest that widespread and rapid clearance of AGAb in wild-type mice takes place throughout the body where the target is expressed, and thus the amount available for neuronal clearance is relatively lower than in GalNAcT^−/−^-*Tg(neuronal)* mice. Conversely, since neurons are the only possible ganglioside-mediated clearance route available in GalNAcT^−/−^-*Tg(neuronal)* mice, the AGAb becomes highly concentrated in the motor neuron and its trafficking pathways. Within the spinal cord 5 days post-immunization, trafficked AGAb was not associated with specific cell types, including neuronal (cell body or dendrite) or glial membranes (Fig. 1G). Other peripherally exposed synapses that would be expected to express gangliosides in GalNAcT^−/−^-*Tg(neuronal)* mice, and thus endocytose antibody include those in post-ganglionic autonomic neurons; these were not examined. Although peripheral sensory nerve terminals were not directly examined, surface deposits of antibody were observed on some dorsal root ganglia cell bodies (Fig. 1H) in the absence of any visible internalized antibody over this time period. No antibody deposits were observed in central sensory connections in the dorsal horn of the spinal cord, rather the AGAb is highly concentrated in the ventral horn cell bodies and surrounding neuropil as would be expected following retrograde trafficking (Fig. 1I). These data suggest that sensory nerve uptake with retrograde trafficking to the dorsal root ganglia is not a major clearance pathway for AGAbs, compared to the extensive contribution of NMJs, although we do not exclude this possibility under other conditions and antibody specificities.

**Figure 2:**
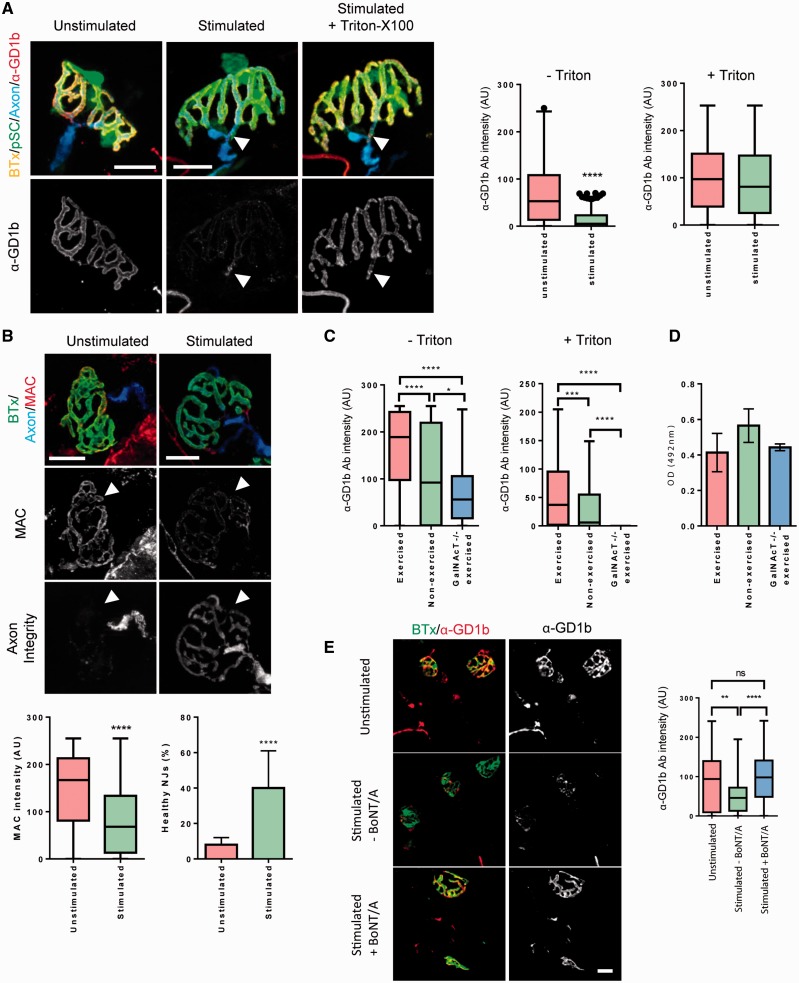
**Uptake at the nerve terminal is activity dependent.** (**A**) Wild-type triangularis sterni muscle (*n *= 3) was labelled with anti-GD1b antibody, then washed and the intercostal nerves of one hemi-triangularis sterni per mouse was stimulated, while the other remained unstimulated. Once analysed, tissue was permeabilized, reprobed with secondary antibody and reanalysed for antibody presence. Unstimulated nerve terminals show a higher presence of membrane anti-GD1b antibody than stimulated nerve terminals. Stimulated nerve terminals still showed presence of anti-GD1b antibody at distal nodes of Ranvier (white arrowhead). Following permeabilization and reprobing with secondary antibody, anti-GD1b intensity in stimulated tissue was no different than unstimulated tissue (^****^P < 0.0001 versus unstimulated, Mann-Whitney test). (**B**) In *ex vivo* preparations, stimulation can protect the nerve terminal from complement-mediated injury. When normal human serum is added to triangularis sterni muscles (*n *= 3), which were labelled with anti-GD1b antibody and subsequently stimulated, deposition of MAC over motor nerve terminals is reduced when compared to unstimulated nerve terminals (^****^P < 0.0001 versus unstimulated, Mann-Whitney test). This is accompanied by retention of cytosolic CFP in the nerve terminal. White arrow head indicates expected position of CFP as indicated by BTx. Fisher’s Exact Test indicated that stimulated triangularis sterni had more healthy NMJs than unstimulated as indicated by the presence of CFP (displayed as mean % of total junctions analysed). ^****^P < 0.0001 versus unstimulated. (**C**) In an acute exercise paradigm, the intensity of anti-GD1b antibody present at whole-mount soleus muscle motor nerve terminals is higher in exercised GalNAcT^−/−^-*Tg(neuronal)* mice compared with unexercised mice (*n *= 3 per group) even after permeabilization and re-probing with secondary antibody. (**D**) In the same exercise paradigm, no significant difference exists between the serum levels of anti-GD1b antibody in exercised and unexercised GalNAcT^−/−^-*Tg(neuronal)* mice. (**E**) In a control experiment (*n *= 1 mouse for unstimulated, stimulated − BoNT/A, *n *= 2 mice for stimulated +BoNT/A, minimum 62 NMJs analysed per group), BoNT/A inhibited the uptake of AGAb caused by stimulation. Scale bars = 20 μm. Non-parametric data are displayed as box and whisker plots.

We next evaluated the effects of neuromuscular synaptic activity on antibody clearance rates in *ex vivo* wild-type triangularis sterni neuromuscular preparations (Fig. 2). Following initial labelling with anti-GD1b AGAb under resting conditions at 4 °C (under which state internalization is limited), upon transfer to room temperature stimulated NMJs showed considerably lower levels of AGAb on their surface membrane than unstimulated NMJs (Fig. 2A). This rapid internalization of surface AGAb was sufficient to reduce MAC deposition and damage to the NMJ membrane in a complement-mediated injury paradigm (Fig. 2B). In an acute exercise paradigm (forced treadmill exercise), the additional motor activity served to increase, rather than decrease the amount of antibody present at the surface of the NMJs of the soleus (lower leg) muscle of GalNAcT^−/−^-*Tg(neuronal)* mice. Intracellular AGAb was also higher due to increased internalization by the active nerve (Fig. 2C). We interpret the increase of AGAb at the surface membrane as being due to increase flow of blood to the exercising muscle, thereby delivering more AGAb. In this acute exercise model (conducted over 3 h) exercising did not detectably change the overall levels of circulating AGAb compared to controls (Fig. 2D). As a further control experiment, BoNT/A was added to block vesicle release (and therefore recycling) in triangularis sterni preparations before stimulation. The addition of BoNT/A attenuated to background levels the clearance of surface AGAb in response to nerve stimulation (Fig. 2E). This reinforces the notion that AGAb uptake is increased by synaptic activity.

**Figure 3:**
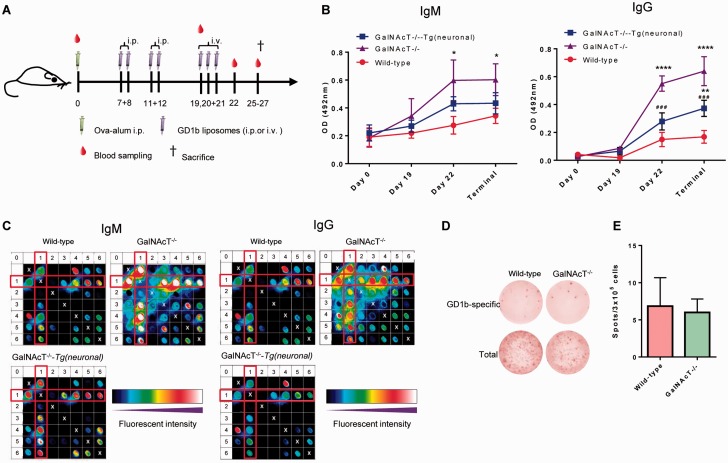
**Serum levels of AGAb in actively immunized mice are attenuated by endocytic clearance.** (**A**) Immunization protocol. (**B**) Anti-GD1b specific ELISA show serum levels of anti-GD1b IgM and IgG were higher in the GalNAcT^−/−^ mice (*n *= 4) than the wild-type mice (*n *= 6), with intermediate values for GalNAcT^−/−^-*Tg(neuronal)* mice (*n *= 6) (*P < 0.05 versus wild-type, two-way ANOVA with Tukey’s multiple comparison test). (**C**) Glycoarray analysis showing illustrative IgM and IgG responses at Day 22 to GD1b and other liposome components both alone and in complex. Arrays are interpreted by reading along both axes to find the resulting combination of lipids, with a line of symmetry marked by white crosses. Numbers represent lipids as follows: 0 = methanol (diagonally), 1 = GD1b, 2 = sphingomyelin, 3 = GM1, 4 = cholesterol, 5 = dicetylphosphate, 6 = GT1b. The GD1b columns are highlighted by a red box. (**D**) Illustrative ELISpot wells show similar spot numbers between GalNAcT^−/−^ and wild-type mice. (**E**) Positive IgG + IgM spots per well for each genotype (+SEM) from wild-type and GalNAcT^−/−^ mouse splenocytes show no significant difference between wild-type (*n *= 4) and GalNAcT^−/−^ mice (*n *= 4) (Student *t*-test).

Self-antigens including gangliosides are considered poor immunogens due to the effects of tolerance in limiting autoimmune responses (Nores *et al.*, 2008). Previous active immunization studies aiming to generate AGAb responses have used GalNAcT^−/−^ mice to bypass tolerance as wild-type mice appear to respond poorly (Lunn *et al.*, 2000; Bowes *et al.*, 2002). As the readout for active immunization is assessment of serum AGAb levels, clearance by neuronal endocytosis could confound interpretation of these studies. To test this, wild-type, GalNAcT^−/−^ and GalNAcT^−/−^-*Tg(neuronal)* mice were serially immunized with GD1b and monitored for antibody production (Fig. 3A). Anti-GD1b titres were highest in GD1b-deficient GalNAcT^−/−^ mice, lowest in wild-type mice and intermediate in GalNAcT^−/−^-*Tg(neuronal)* mice by ELISA (Fig. 3B). Serum from GalNAcT^−/−^ mice also showed the highest fluorescent signal to GD1b by glycoarray, both alone and in complex with other lipids (Fig. 3C). These data indicate that either tolerance or clearance is regulating AGAb levels in wild-type mice. To examine this, we assessed GD1b antigen-specific B cell frequency by ELISpot assays and found comparable numbers of GD1b-specific B cells (combined IgG and IgM) in wild-type and GalNAcT^−/−^ mice (Fig. 3D and E). These data indicate that wild-type and GalNAcT^−/−^mice equally respond to ganglioside immunization, but that the former prematurely deplete serum AGAb by endocytosis. GalNAcT^−/−^-*Tg(neuronal)* mice most likely respond to immunization similarly to other genotypes, but clear AGAb solely though neuronal endocytosis. We were unable to detect endocytosed IgG in the motor axon, cell body or spinal cord of actively immunized GalNAcT^−/−^-*Tg(neuronal)* mice, likely due to levels being below the detection threshold.

## Discussion

**Figure 4:**
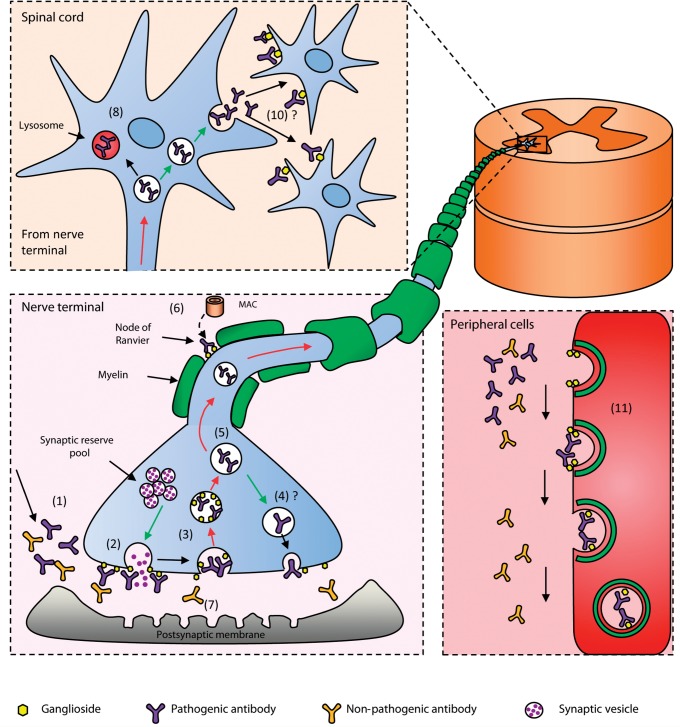
**Schematic representation of anti-ganglioside antibody internalization.** At the nerve terminal, pathogenic and non-pathogenic anti-ganglioside antibodies (that can and cannot bind their ganglioside target when embedded in the plasma membrane, respectively) arrive from the circulation (**1**). Vesicles containing ACh fuse with the membrane during neurotransmitter release and during activity-independent vesicle recycling (**2**), pathogenic antibody species bind to gangliosides. During vesicle retrieval, gangliosides and their attached antibodies are internalized (**3**). Antibodies may be directly recycled to the plasma membrane (**4**), or undergo retrograde axonal transport to the motor neuron cell body in the spinal cord (**5**). Pathogenic anti-ganglioside antibody, which also binds at the node of Ranvier, cannot be significantly internalized, and therefore activate complement, resulting in MAC deposition and nodal injury (**6**). Non-pathogenic antibodies are unbound and freely circulate in the interstitial environment of the nerve terminal and are not detectably internalized (**7**). Retrogradely transported antibody residing in the soma of motor neurons may be degraded in lysosomes (**8**), or recycled to the neuron surface (**9**) and secreted, free to bind to adjacent neurons and potentially mediate pathogenic effects (**10**). Elsewhere in the body, circulating pathogenic antibodies may bind to their target ganglioside in the membrane of non-neuronal cells. Once bound, antibodies may be endocytosed thus cleared from the circulation (**11**), again leaving only non-pathogenic antibodies to circulate in the serum. These will be the preferential antibody species detected by solid-phase assays such as ELISA. Exocytic pathways are shown by green arrows while endocytic pathways are shown by red arrows.

We have identified a major clearance pathway for AGAbs that are harvested by peripheral nerve endocytosis (Fig. 4). Estimating the surface area of the human NMJ presynaptic membrane at ∼0.1 m^2^ (Supplementary material), this represents a sizable sink for depleting circulating IgG and other factors that bind this endocytically active membrane. The local and/or distant neurotoxic effects of internalized and retrogradely transported AGAb are unknown, as are the onward trafficking and catabolic pathways, but could mediate CNS manifestations of Guillain-Barré syndrome variants. Inhibitory interneuron damage, as seen in tetanus toxicity, could be implicated in the hyper-reflexia seen in AGAb-associated AMAN (acute motor axonal neuropathy) cases (Kuwabara *et al.*, 1999; Yuki *et al.*, 2012). Similarly, injury to the reticular activating system adjacent to oculomotor nuclei in the brainstem from endocytosed AGAb may underlie the coma seen in the Bickerstaff’s brainstem encephalitis variant of Guillain-Barreé syndrome (Shahrizaila and Yuki, 2013). Antibodies against unidentified synaptic membrane components have previously been shown to translocate from spinal cord neurons following retrograde trafficking from the periphery (Wenthold *et al.*, 1984; Ritchie *et al.*, 1986; Fabian, 1988). Most studies reporting ingress of autoantibodies into the CNS assume they are delivered through the blood–brain barrier (Wootla *et al.*, 2015; Xu *et al.*, 2015). However peripheral neuron uptake merits further consideration. Equally, ganglioside binding proteins, such as α-synuclein and amyloid-β peptides could be delivered from the systemic circulation to the CNS through a NMJ uptake route (Hoshino *et al.*, 2013; Sacino *et al.*, 2014).

Endocytic clearance of circulating antibody by self-antigen confounds interpretation of studies dependent upon measuring serum antibody levels. Not all AGAbs are neurotoxic as exemplified in this study by using the anti-GM1 antibodies DG1 and DG2. During polyclonal AGAb responses, non-tissue binding AGAbs that cannot be cleared by endocytosis will predominate in the circulation over tissue binding AGAbs, thereby misrepresenting the diversity of the AGAb repertoire. In neuropathy research, harmless AGAbs will be the predominant circulating species, thereby confounding pathogenesis studies that rely on demonstrating neurotoxic effect with human or animal sera (Greenshields *et al.*, 2009; Galban-Horcajo *et al.*, 2014). These data help to resolve longstanding enigmas surrounding AGAb in the context of immune homeostasis and disease, in addition to raising new clinical and basic research questions.

## Funding

R.M., J.A.B., D.Y and H.J.W. are supported The Wellcome Trust Grant 092805. M.E.C. was supported by a Medical Research Council PhD studentship. G.R.M. was supported by a Guillain-Barré Syndrome Support Group UK studentship.

## Supplementary material

Supplementary material is available at *Brain* online.

## Supplementary Material

Supplementary Data

## Abbreviations

AGAbs =: anti-ganglioside antibodies
BoNT/A =: Botulinum toxin A
NMJ =: neuromuscular junction
