# Supplementary material for: Anti-ganglioside antibodies are removed from circulation in mice by neuronal endocytosis
Source: Brain. 2016 Mar 26;139(6):1657–65. doi: 10.1093/brain/aww056 (PMC4892750; doi:10.1093/brain/aww056)
Supplement: Supplementary Data [file aww056_supplementary_data.zip › brain-2015-02124-File008.pdf]

Supplementary Material

Staining of cervical cord. Sections of 15 µm thickness were washed 3x with 0.5% Triton X-100/PBS then blocked for 1h at room temperature in 3% NGS/PBS. Rabbit anti-mouse NeuN *(Millipore, Billerica, MA, USA)* (1/750), rabbit anti-mouse Neurofilament heavy *(Catalogue number BML-NA1211-0050, Enzo Life Sciences, Exeter, UK)* (1/200), rabbit anti-mouse GFAP *(catalogue number Z0334, Dako, Glostrup, Denmark),* (1/1000) or rat anti-mouse MBP *(Clone aa82-87 AbD Serotec, Oxford, UK)* (1/500) were applied overnight at 4ºC in 3% NGS/PBS. The following day, appropriate TRITC-conjugated secondary antibodies *(Southern Biotech, Birmingham, AL, USA)* were applied (5 µg/ml in PBS with 1% NGS) for 3 hours at 4ºC. Tissue was washed three times in PBS, mounted in Vectashield (with DAPI), *(Vector Laboratories, Burlington, Canada)* and stored at -20ºC until imaged.

Botulinum toxin intoxication

triangularis sterni muscle was dissected as in Methods. Muscle was incubated in BoNT/A (Dysport®, Ipsen biopharmaceuticals) at a concentration of 200 U/ml in Ringer's solution for 4 hours at room temperature before stimulation as described in methods.

Exercise studies.

GalNAcT$^{-/-}$-*Tg(neuronal)* mice were injected with 250µg, anti-GD1b antibody, then placed on a treadmill. Exercised mice were warmed up with a treadmill speed of 0.04 m/sec, increased by 0.2 m/sec every 2 minutes up to 0.1 m/sec. Speed was maintained for 15 minutes before increasing by 0.2 m/sec every 2 minutes until 0.22 m/sec. This speed was maintained for 30 minutes. Treadmill was slowed to a stop and mice given 30 minute rest period before repeating the steps three times. Non-exercised mice remained on a stationary treadmill. Mice were culled immediately. All groups of mice were trained using the test protocol for 3 days the week prior to the experiment.

Glycoarray methodology.

Using combinatorial glycoarray (Rinaldi *et al.*, 2009) serum and monoclonal antibodies were tested against single lipids and lipids complexes. Slides were pre-prepared by applying Immobilon-FL PVDF membrane *(Merk Millipore, Billerica, MA, USA)* to glass slides using photomount glue *(3M, MN, USA)*. Once glue was dry, 0.1 µl of each lipid or premixed lipid complex (100 µg/ml for single lipid, 50 µg /ml for each of the lipids in complex) was spotted using an automatic TLC sampler *(CAMAG, Muttenz, Switzerland),* onto the slides. Once spotted, slides were left overnight at 4°C then blocked with 2% BSA for 1 hour at room temperature. Serum (1/100 in 1% BSA) was applied to slides for 1 hour at 4°C. Following 2x 15 minute washes in 1% BSA, goat anti-mouse IgG or IgM Alexa Fluor 555 or 647-conjugated antibody was applied (2 µg/ml in 1% BSA) for 1 hour at 4°C. Slides were washed 2x 30 minutes in 1% BSA, followed by 1x 5 minutes wash with PBS and 1x 5 minute wash with dH2O. Slides were left to dry overnight at 4°C then scanned using a ScanArray express microarray scanner *(Perkin-Elmer, Waltham, MA, USA).*

ELISpot methodology.

MultiScreen-IP Filter Plates (*Merk Millipore, Billerica, MA, USA*) were activated with 35 µl 35% ethanol for 30 seconds. Ethanol was discarded and plates coated with 100 µl of 50 µg/ml GD1b in 100% ethanol, or ethanol only for blank wells. When ethanol had evaporated, plates were blocked with RPMI with 10% FCS for 1 hour in a CO_2 incubator at 37°C. Block was discarded and 100 µl previously isolated splenocytes were added at $3x10^5$ cells per well, overnight in a CO_2 incubator at 37°C. The following day cells were discarded and plates washed in PBS before incubating with secondary antibody (HRP conjugated goat anti-mouse IgG and IgM) for 1 hour at 4°C. Secondary was discarded and plates washed a further four times in PBS. Plates were developed by incubation with 3-Amino-9-ethylcarbazole (AEC) substrate

(Sigma-Aldrich, St Louis, MO, USA). The AEC substrate solution (100 µl) was added to wells for around 20 minutes, or until spots began to develop. Reaction was stopped by washing wells with dH_2O. Wells were imaged using an AID ELISpot reader *(Autoimmun Diagnostika GmbH, Strassberg, Germany)* and spots were counted using the provided AID software.

Estimation of pre-synaptic membrane total surface area in man. The number of muscle fibres in man was taken from a previous estimate of $2.5x10^8$ calculated by Howell and Fulton, 1949 and critically discussed by Bianconi et al (Bianconi *et al.*, 2013). The average surface area of the presynaptic membrane was estimated at 400 square microns. Therefore the total surface area in is estimated at $2.5x10^8$ x 400 square micron = $10x10^{10}$ square microns = 0.1 square metres.

Box and whisker Plots

Box and whisker plots show the spread of data from all NMJs or NeuN-positive cells analysed per treatment. The horizontal line displays the median of the data, with the top and bottom of the box representing the 75th and 25th percentile respectively. Top whiskers denote the 75th percentile plus 1.5x the interquartile range (IQR). Bottom whiskers denote the 25th percentile minus 1.5x the IQR and any values outwith this range are plotted as individual points.
