# Supplementary figures and images for: Anti-ganglioside antibodies are removed from circulation in mice by neuronal endocytosis
Source: Brain. 2016 Mar 26;139(6):1657–65. doi: 10.1093/brain/aww056 (PMC4892750; doi:10.1093/brain/aww056)

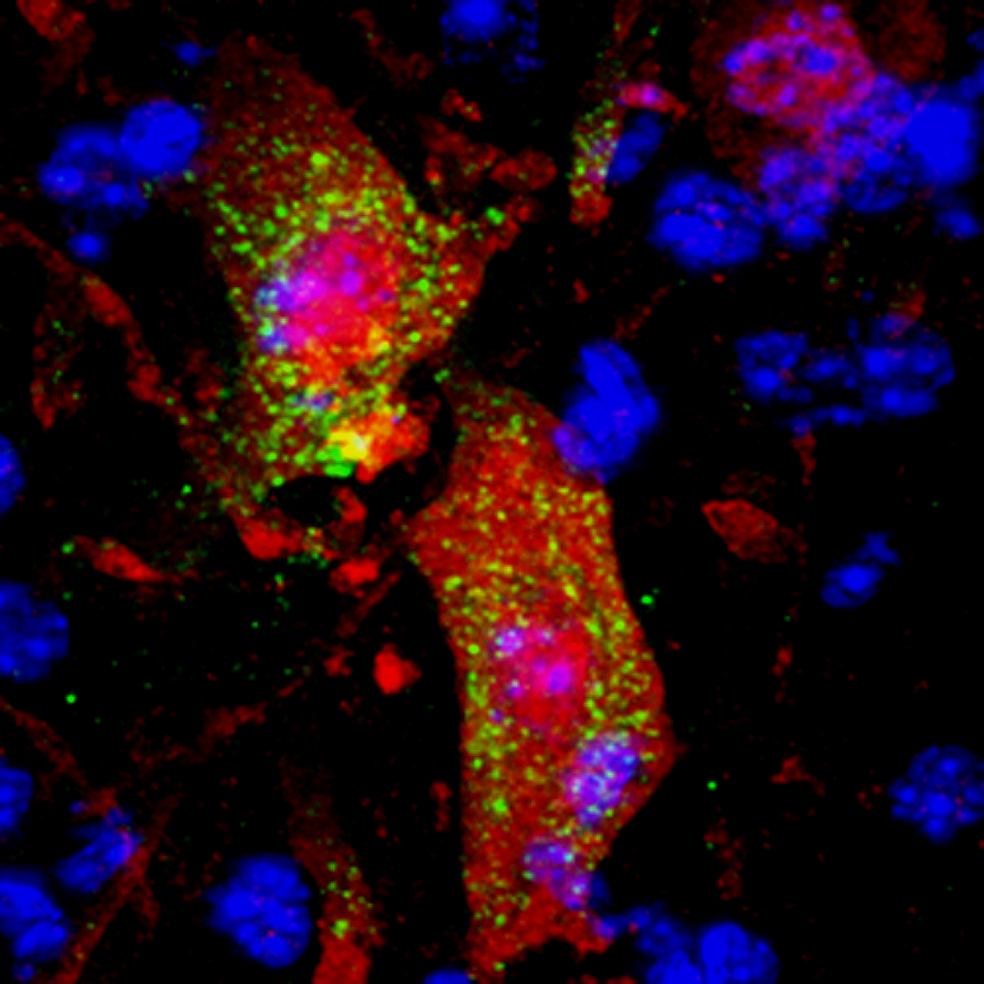

Supplement: Supplementary Data [file aww056_supplementary_data.zip › brain-2015-02124-File006.jpg]
